# Supplementary material for: Peptides Derived from (RRWQWRMKKLG)2-K-Ahx Induce Selective Cellular Death in Breast Cancer Cell Lines through Apoptotic Pathway
Source: Int J Mol Sci. 2020 Jun 26;21(12):4550. doi: 10.3390/ijms21124550 (PMC7352952; doi:10.3390/ijms21124550)
Supplement: Supplementary file 1 [file ijms-21-04550-s001.pdf]

International Journal of *Molecular Sciences*

MDPI

Supplementary Materials

Peptides Derived from (RRWQWRMKKLG)$_2$-K-Ahx Induce Selective Cellular Death in Breast Cancer Cell Lines through Apoptotic Pathway

Diego Sebastián Insuasty-Cepeda[1], Andrea Carolina Barragán-Cárdenas[1], Alejandra Ochoa-Zarzosa[2], Joel E. López-Meza[2], Ricardo Fierro-Medina[1], Javier Eduardo García-Castañeda[3] and Zuly Jenny Rivera-Monroy[1,*]

* Correspondence: zjriveram@unal.edu.co; Tel.: +57-1-3165000 (ext. 14436)

Analysis by RP-HPLC of peptide 26[M] after four months of storage.

Dimer **^{26}[M]: (RRWQWRMKKLG)$_2$-K-*Ahx*,** (10 μL, 1 mg/mL) was analyzed, at different times of storage, by RP-HPLC using an Agilent Series 1260 chromatograph; Chromolith® C-18 monolithic column (50 × 4.6 mm). Solvent A: 0.05% TFA in water, Solvent B: 0.05% TFA in ACN, for the analysis an elution gradient of 5 to 50% of solvent B was used, gradient time was 8 minutes at a flow rate of 2.0 mL/min, and 210 nm for detection.

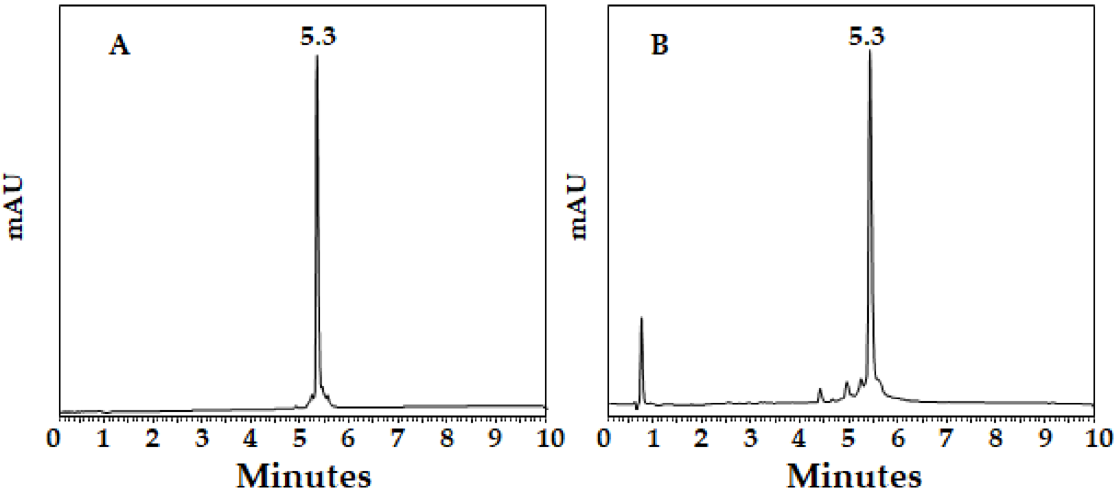

Figure S1. Analysis by RP-HPLC of purified peptide ^{26}[M]: (RRWQWR**M**KKLG)$_2$-K-*Ahx* at zero time (A) and after four months of storage (B).

Synthesis of the dimeric peptides by SPPS-Fmoc/tBu

The dimeric peptides (RRWQWR**M**KKLG)$_2$-K-Ahx (^{26}[M]) and its analogs were synthesized using the solid phase peptide synthesis (SPPS) methodology by the Fmoc/tBu strategy. The Rink Amide resin (0.46 meq/g, Figure S2a) was used as solid support, this resin (200 mg) was conditioned with DMF for 12 h. Figure S2 shows the general scheme for obtaining the dimeric peptides, please note that in red is shown the 26th residue that was Methionine for the original peptide, ^{26}[M], and it was replaced by Lys, Asp, Ala, Phe or Leu for the dimers ^{26}[K], ^{26}[D], ^{26}[A], ^{26}[F] and ^{26}[L], respectively.

Briefly, SPPS-Fmoc/tBu involved three steps [48,49], Figure S2. (Step 1) The Fmoc group removal: this reaction was carried out by treating the protected resin with 4-methylpiperidine (2.5% v/v) in DMF, 10 min at room temperature (RT) (×2). (Step 2) The Fmoc-amino acid coupling: for attaching each amino acid, the corresponding Fmoc-amino acid (0.46 mmol) was first threated with DCC/6-Cl-HOBt (0.45/0.46 mmol) in DMF, the reaction was stirred vigorously for 15 min at RT. This mixture was then added to the unprotected resin and allowed to react for 1 h, with stirring. The Kaiser test was performed to monitor both the Fmoc removal and coupling reaction. For obtaining each dimer, the amino acid Fmoc-6-aminohexanoic (*Ahx*) was bound as the C-terminal residue (Figure S2c-d), then Fmoc-Lys(Fmoc)-OH amino acid was attached (Figure S2e), and by Fmoc removal reaction it was generated two free amino group, at *α* and ε position, and over them the target sequence was constructed, generating a protected dimeric structure (Figure S2f). (Step 3) Cleavage reaction: the side chains protecting groups removal and the peptide cleavage from the resin was carried out by treating the dried resin-peptide with a solution containing TFA/H_2O/TIS/EDT (9.5/2.5/2.5/2.5%, v/v), the reaction was stirred for 4 h at RT. Then, the resin was filtered and the solution was treated with cold ethyl ether precipitating the dimer. Finally, crude products were purified by SPE [50] and analyzed by RP-HPLC and MALDI-TOF MS.

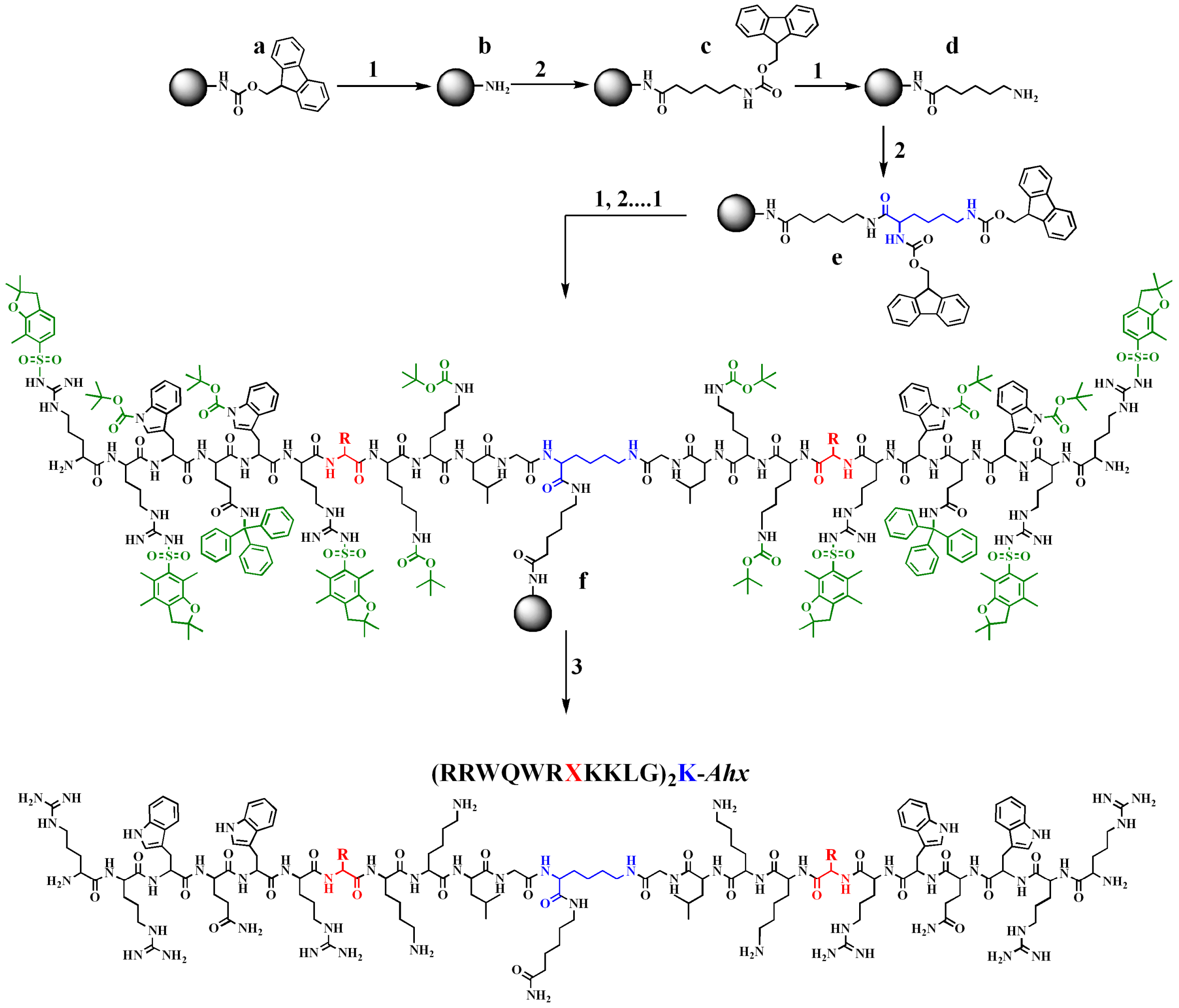

Figure S2. Synthesis of dimeric peptides by SPPS-Fmoc/*t*Bu. Lysine residue used for building the two branches, in blue. The amino acid at position 26, in red. The protecting groups of the side chain from the amino acids, in green. 1: The Fmoc group removal. 2: The Fmoc-amino acid coupling. 3. Cleavage reaction.

Purified Dimer Characterization by RP-HPLC and MALDI-TOF MS

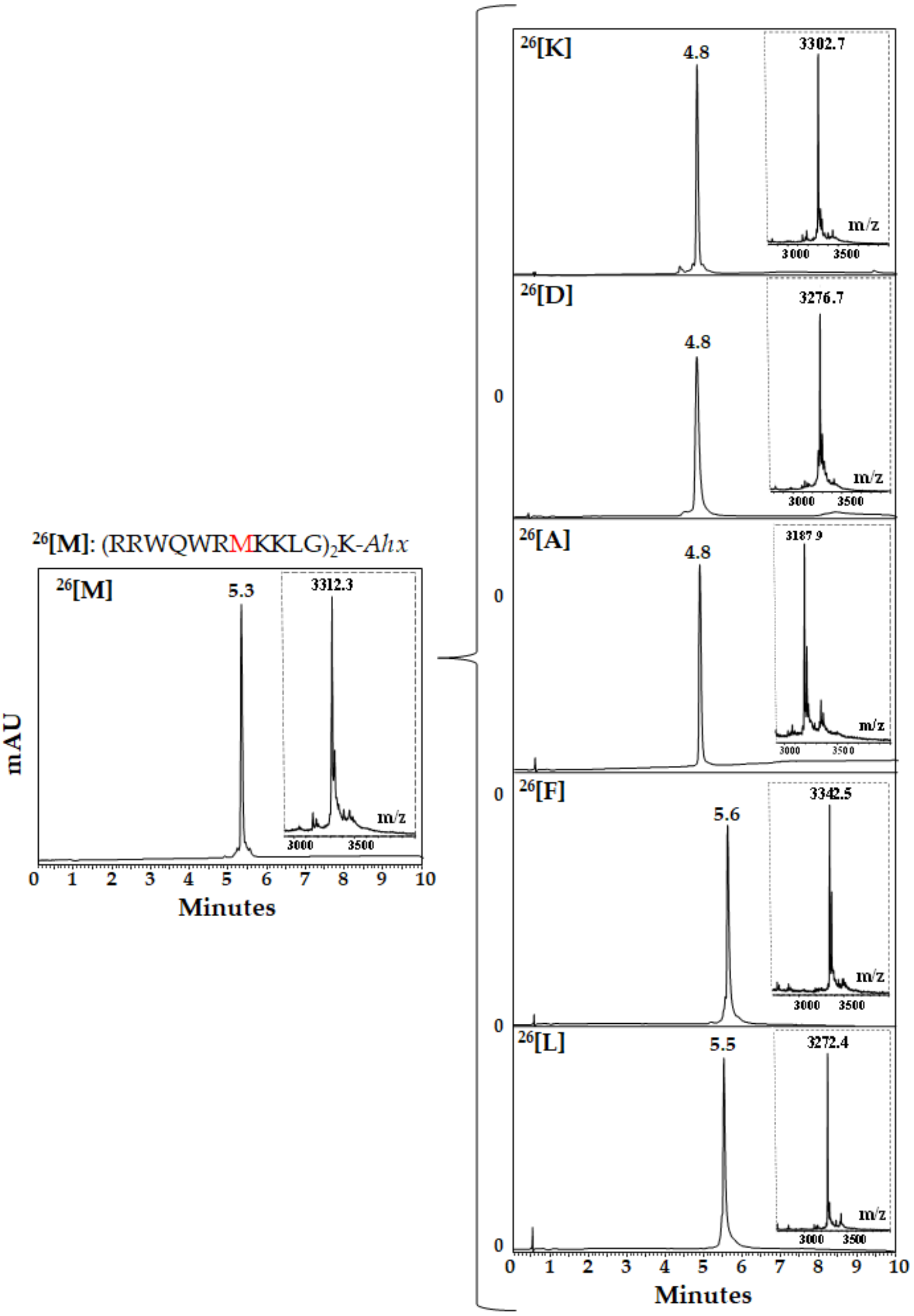

Figure S3. Characterization of purified (RRWQWR**M**KKLG)$_2$-K-*Ahx* (^{26}[M]) and its analogs. Chromatographic profiles and MALDI-TOF MS spectra (in dashed square). The residue at position 26 corresponds to Lys, Asp, Ala, Phe or Leu for ^{26}[K], ^{26}[D], ^{26}[A], ^{26}[F] and ^{26}[L], respectively. Peptides were analyzed as described above and in Materials and Methods

© 2020 by the authors. Submitted for possible open access publication under the terms and conditions of the Creative Commons Attribution (CC BY) license (http://creativecommons.org/licenses/by/4.0/).
